# Reactive, Adult Neurogenesis From Increased Neural Progenitor Cell Proliferation Following Alcohol Dependence in Female Rats

**DOI:** 10.3389/fnins.2021.689601

**Published:** 2021-09-14

**Authors:** Natalie N. Nawarawong, K. Ryan Thompson, Steven P. Guerin, Chinchusha Anasooya Shaji, Hui Peng, Kimberly Nixon

**Affiliations:** ^1^College of Pharmacy, The University of Texas at Austin, Austin, TX, United States; ^2^Division of Pharmacology & Toxicology, College of Pharmacy, The University of Texas at Austin, Austin, TX, United States

**Keywords:** alcohol, ethanol, hippocampus, abstinence, recovery, alcoholism, adult neurogenesis, neural stem cells

## Abstract

Hippocampal neurodegeneration is a consequence of excessive alcohol drinking in alcohol use disorders (AUDs), however, recent studies suggest that females may be more susceptible to alcohol-induced brain damage. Adult hippocampal neurogenesis is now well accepted to contribute to hippocampal integrity and is known to be affected by alcohol in humans as well as in animal models of AUDs. In male rats, a reactive increase in adult hippocampal neurogenesis has been observed during abstinence from alcohol dependence, a phenomenon that may underlie recovery of hippocampal structure and function. It is unknown whether reactive neurogenesis occurs in females. Therefore, adult female rats were exposed to a 4-day binge model of alcohol dependence followed by 7 or 14 days of abstinence. Immunohistochemistry (IHC) was used to assess neural progenitor cell (NPC) proliferation (BrdU and Ki67), the percentage of increased NPC activation (Sox2+/Ki67+), the number of immature neurons (NeuroD1), and ectopic dentate gyrus granule cells (Prox1). On day seven of abstinence, ethanol-treated females showed a significant increase in BrdU+ and Ki67+ cells in the subgranular zone of the dentate gyrus (SGZ), as well as greater activation of NPCs (Sox2+/Ki67+) into active cycling. At day 14 of abstinence, there was a significant increase in the number of immature neurons (NeuroD1+) though no evidence of ectopic neurogenesis according to either NeuroD1 or Prox1 immunoreactivity. Altogether, these data suggest that alcohol dependence produces similar reactive increases in NPC proliferation and adult neurogenesis. Thus, reactive, adult neurogenesis may be a means of recovery for the hippocampus after alcohol dependence in females.

## Introduction

Alcohol misuse is a leading cause of preventable death due to its toxic consequences across multiple organs of the body (Centers for Disease Control and Prevention [CDC], 2021). Alcohol use disorder (AUD), for which the hallmark symptom is the excessive consumption of alcohol despite negative consequences or harm, is diagnosed in nearly 14% of United States adults in any given year (Grant et al., 2015). While the lifetime prevalence of a severe AUD in women is about half (9.7%) of what is reported for males (18.3%), over the last decade, the annual AUD diagnosis rate has increased 84% in women compared to only 35% in men (Grant et al., 2017; Fama et al., 2020). Thus, the gender gap is narrowing across a variety of alcohol-related metrics (Keyes et al., 2008; Grant et al., 2015; Grucza et al., 2018; White, 2020), but few studies have examined only females, especially in preclinical models.

Women appear to be more vulnerable to the damaging effects of alcohol in various organ systems when compared to men, even when accounting for fewer years of harmful consumption or dependence (Nixon et al., 2014; Fama et al., 2020). Clinical studies have found that despite a shorter history of alcohol dependence, females exhibit similar levels of brain degeneration as males, which suggests that they are more vulnerable to the neurotoxic effects of alcohol (Mann et al., 1992, 2005). The brain is especially impacted by the toxic effects of alcohol with detectable cognitive impairments across learning, memory, executive function, and motor processes related to gait and balance (Sullivan, 2000a,b; Crews and Nixon, 2009; Nixon et al., 2014; Cortez et al., 2020; Fama et al., 2020). However, little is known about the damaging effects of alcohol on the female brain. Many studies show that women develop alcohol-induced brain damage faster than males despite decreased use and misuse (Crawford and Ryder, 1986; Jacobson, 1986; Nixon et al., 1995, 2014; Flannery et al., 2007; Fama et al., 2020; White, 2020) though a few fail to find a difference between males and females in hippocampal volume loss (Demirakca et al., 2011; Grace et al., 2021). Interestingly, with abstinence from alcohol drinking, brain and behavioral recovery have been observed (Carlen et al., 1978; Jacobson, 1986; Pfefferbaum et al., 1995; Sullivan et al., 2000; Gazdzinski et al., 2005; Demirakca et al., 2011; Hoefer et al., 2014; Zou et al., 2018). Females may exhibit quicker structural brain recovery and withdrawal symptom cessation (Jacobson, 1986; Deshmukh et al., 2003), but they may suffer from more prolonged cognitive impairment compared to males (Luquiens et al., 2019).

The hippocampus is one brain region that is both a target of alcohol’s neurotoxic effects (Sullivan et al., 1995; Wilson et al., 2017), but also has the ability to recover with abstinence (Sullivan et al., 2000; Bartels et al., 2007; Hoefer et al., 2014). Clinical studies have observed recovery of hippocampal volume loss due to alcohol use as well as improved cognitive function, as assessed with learning and memory, intelligence, and attention tasks, with both short-term and extended periods of abstinence (Brandt et al., 1983; Bartels et al., 2007; Demirakca et al., 2011; Hoefer et al., 2014; Zou et al., 2018). While the mechanisms underlying brain recovery during abstinence remain unknown, many purport that it may not be mere coincidence that hippocampal recovery coincides with the presence of neural stem cells (NSCs) and the ongoing generation of new neurons, or adult neurogenesis (Nixon, 2006; Mandyam and Koob, 2012; Geil et al., 2014; Toda et al., 2019).

Neurogenesis from NSCs contributes to both hippocampal structure and function throughout the life of an organism (see Toda et al., 2019; Snyder and Drew, 2020 for review). Asymmetrical division of NSCs that reside within the subgranular zone (SGZ) of the dentate gyrus generate NPCs that differentiate and ultimately mature into newborn granule cells (Palmer et al., 1997). Current theories suggest that true NSCs of the neurogenic niche are radial glial-like cells that are largely quiescent and express the Sox2 transcription factor as well as astrocyte markers, such as glial fibrillary acidic protein (GFAP; Bonaguidi et al., 2011). Activated NSCs, those that express the endogenous cellular proliferation marker Ki67, give rise to intermediate NPCs, which divide rapidly and birth more fate-restricted NPCs that eventually become mature granule cells (Seri et al., 2001; Kronenberg et al., 2003). While neurogenesis continues throughout the lifespan of most mammals studied to date (Eriksson et al., 1998; Spalding et al., 2013; Boldrini et al., 2018), there is a lingering debate as to the extent and role of these cells in humans (Boldrini et al., 2018; Sorrells et al., 2018; for review see Kumar et al., 2019). Preclinical studies have shown that adult neurogenesis is necessary for hippocampal functioning, with reductions associated with impairments in function, while conversely, increasing adult neurogenesis is associated with improvements, such as better learning and memory performance (Shors et al., 2001; Imayoshi et al., 2008; Clelland et al., 2009; Sahay et al., 2011). Indeed, alcohol intoxication concentration-dependently reduces NPC proliferation and neurogenesis in adolescent and adult organisms, including humans (Nixon and Crews, 2002, 2004; Herrera et al., 2003; Crews et al., 2006; Richardson et al., 2009; Golub et al., 2015; Le Maitre et al., 2018). Injury-induced neurogenesis, often referred to as “reactive neurogenesis” has been observed in rodents after several forms of brain insult such as traumatic brain injury (Dash et al., 2001; Blaiss et al., 2011; Yu et al., 2016), ischemia (Jin et al., 2006), seizure (Parent et al., 1997; Scharfman et al., 2003), and notably upon abstinence after alcohol dependence (Nixon and Crews, 2004; Somkuwar et al., 2016; West et al., 2019). However, whether reactive neurogenesis is beneficial and reparative is still up for debate (Blaiss et al., 2011; Cho et al., 2015; Golub et al., 2015; Yu et al., 2016; Lee et al., 2019; Nickell et al., 2020). Recent work in females suggests that they may exhibit reactive neurogenesis during abstinence from alcohol as has been observed previously in males (Maynard and Leasure, 2013; Maynard et al., 2017; West et al., 2019). However, whether increased neurogenesis occurs and whether this is due to increased NPC activation has yet to be studied in female rodent models of AUD to date. Furthermore, astrocytic dysfunction has been observed in females but not males (Wilhelm et al., 2016). With the known relationship between astrocytes and NSCs and NPCs, this suggests that there could be a sex specific effect of alcohol on adult NPC-related phenomena during abstinence.

In this study, we investigated the effects of alcohol dependence on NPCs in the dentate gyrus of the hippocampus and its impact on cell cycle activation during recovery in abstinence. Previous studies report a reactive increase in NPC proliferation in the SGZ of the dentate gyrus following 1 week of abstinence from alcohol dependence in male rats (Nixon and Crews, 2004; Hayes et al., 2018). These increases in proliferation result in significantly more immature neurons in the SGZ 14 days after the cessation of binge alcohol exposure according to immature neuron markers Doublecortin (Nixon and Crews, 2004) and NeuroD1 (Hayes et al., 2018). However, these investigations focused on alcohol-induced reactive neurogenesis in males alone, with little known about alcohol effects on adult neurogenesis, let alone reactive neurogenesis, in female preclinical models. Therefore, the aim of this study was to examine NPC proliferation and neurogenesis in females after alcohol dependence, using identical methodologies as those employed in males. As females may be more susceptible to the negative effects of alcohol, we hypothesized that the reactive changes in NPC and adult-born neurons observed in males would also be apparent in females.

## Materials and Methods

### Animal Model of an AUD

A total of 30 adult female Sprague-Dawley rats (∼235 g, Charles River Laboratories, Raleigh, NC, United States) were used across two institutions [University of Kentucky (UK) *N* = 18, Con = 10, and EtOH = 8; The University of Texas at Austin (UT) *N* = 12, Con = 6, and EtOH = 6]. Rats were age matched to prior work in males (9–10 weeks of age) as females weigh less than males. Although the animal model was performed in two different locations, the experimental procedures were identical following Morris et al., 2010b and some personnel were identical between both universities. Rats were allowed to acclimate to either the University of Kentucky or The University of Texas at Austin AAALAC-accredited vivariums for 5 days before the start of experimental procedures. Rats were double-housed in standard polycarbonate cages (all UT, some UK) or polysulfone cages (most UK cages) during all phases of the study except for 24 h during withdrawal scoring. All rats were maintained on a 12-h light/dark cycle (lights on 0700-1900) and allowed *ad libitum* access to standard, 18% protein rodent chow (UK = Teklad 2018 diet, Envigo, Madison, WI, United States; UT = Prolab^®^ RMH 1800 5LL2^∗^, LabDiet, St Louis, MO, United States) and water (UK mostly polysulfone bottles, UT = polycarbonate bottles) for the duration of the experiment apart from the 4 days of binge treatment when food was removed. Prior to the binge exposure, all rats were handled individually for 3 min per day for three consecutive days. All experimental procedures were approved by both the University of Kentucky and The University of Texas at Austin Institutional Animal Care and Use Committees and followed the NIH guidelines for the Care and Use of Laboratory Animals.

**FIGURE 1:**
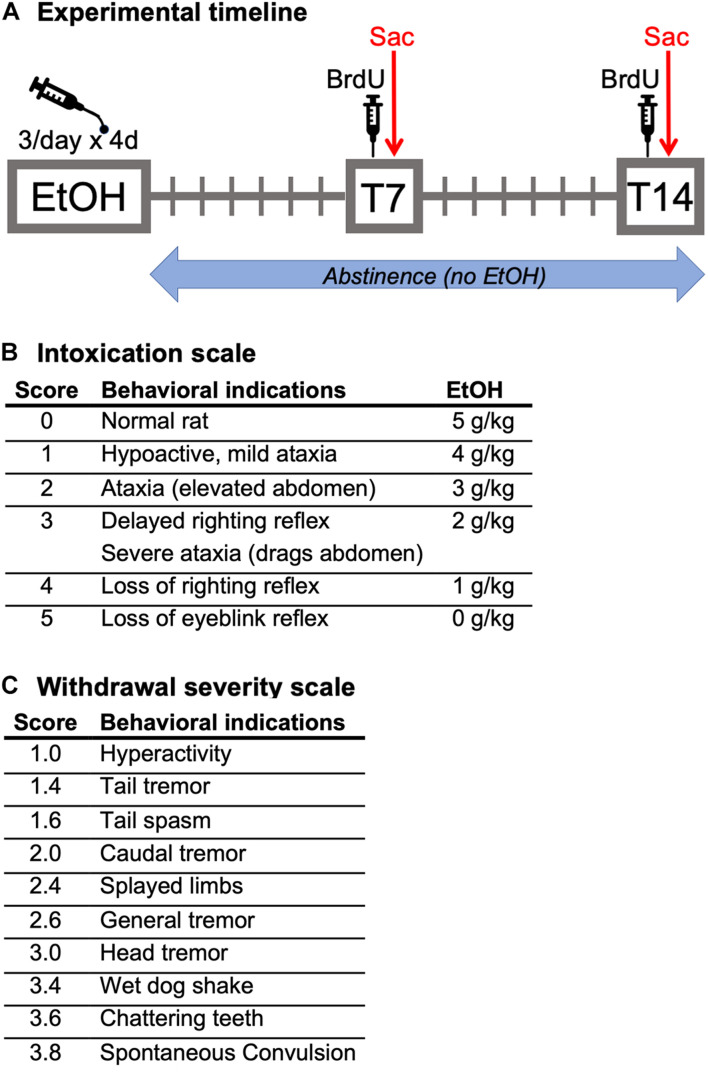
**(A)** Experimental timeline: Following a 4-day binge alcohol exposure, female rats underwent abstinence for either 7 (BrdU, Ki67, Sox2, and Sox2/KI67 analysis) or 14 (BrdU, Ki67, NeuroD1, and Prox1 analysis) days in the home cage. Two hours prior to perfusion at both time points, all rats were injected with BrdU. **(B)** Intoxication behavior scale and dosing. **(C)** Withdrawal severity scale.

Adult female Sprague-Dawley rats were treated with ethanol *via* intragastric gavage in a binge model of an AUD (Majchrowicz, 1975; Morris et al., 2010). As shown in the experimental timeline (Figure 1A), ethanol (25% w/v in Vanilla Ensure Plus^®^, Abbott Labs, Columbus, OH, United States) was administered 3 times a day for 4 days (3 pm, 11 pm, and 7 am). Rats received an initial 5.0 g/kg dose of ethanol with subsequent doses determined based on the behavioral intoxication of the rat (Figure 1B). Control rats were run simultaneously with ethanol-treated rats. Control rats received an isocaloric diet of Vanilla Ensure Plus^®^ containing dextrose, the volume of which was an average of that received by all ethanol-exposed rats for each session. For blood ethanol concentration (BEC) determination, tail blood was drawn using heparinized capillary tubes on day 3 of the binge (90 min after the seventh binge dose) and stored in microcentrifuge tubes on ice containing 3 μl of heparin. After all blood samples were collected, they were centrifuged for 5 min at 6,500 rpm, and stored at −20°C until processing. BECs were measured from plasma by an AM1 Alcohol Analyser (Analox Instruments, Lunenburg, MA, United States). All blood samples were run in triplicate. Withdrawal severity was measured by scoring withdrawal behaviors in the home cage every 30 min, beginning 10 h after the final dose of alcohol (5 pm) to hour 26 (9 am; 16 h). Withdrawal behavior was scored exactly as previously described (Figure 1C; Morris et al., 2010b). For each 30-min block, the most severe observed behavior is recorded, and a mean withdrawal score was determined by averaging the scores from all the observed time points for each rat. Peak withdrawal score is the most severe withdrawal behavior detected throughout the entire observation period.

### Tissue Preparation

As prior work in males has shown peak increases in NPC proliferation on the seventh day of abstinence from a 4-day binge ethanol exposure followed by increases in immature neuron markers 1 week later (Nixon and Crews, 2004; Hayes et al., 2018), rats were sacrificed either 7 (T7) or 14 (T14) days after the final dose of alcohol. To identify dividing cells the thymidine analog, bromodeoxyuridine (BrdU 300 mg/kg; i.p.; Millipore Sigma, Burlington, MA, United States; Cameron and McKay, 2001) was injected at T7 (*n* = 18) or T14 (*n* = 12). Rats were sacrificed two h following BrdU injections with a lethal dose of sodium pentobarbital (i.p.; Fatal-Plus^®^, Vortech Pharmaceuticals, Dearborn, MI, United States) and transcardially perfused using 0.1 M phosphate buffered saline (PBS) followed by 4% paraformaldehyde (PFA). Brains were removed and post-fixed for 24 h in 4% PFA, then stored in PBS pH 7.4 (Gibco, Life Technologies, Grand Island, NY, United States) until sectioning. Unbiased tissue collection methodologies were employed, identical to previous studies (McClain et al., 2011; Hayes et al., 2018; Zhao and van Praag, 2020). Twelve series of coronal sections were cut at 40 μm on a vibrating microtome (Leica VT1000S, Wetzlar, Germany) starting randomly mid striatum through the caudal extent of brain tissue. Sections were stored in series, in 24-well plates with a cryoprotectant at −20°C until histological processing.

### Immunohistochemistry

In order to establish whether reactive neurogenesis occurs in females during abstinence from alcohol, we examined multiple neurogenesis-related markers *via* Immunohistochemistry (IHC) as reported in prior work in male rats (Nixon and Crews, 2004; McClain et al., 2011, 2014; Hayes et al., 2018). First, BrdU IHC was used to identify dividing cells in S-phase, which is confirmed by Ki67 IHC, an endogenous marker of cell proliferation (Kee et al., 2002; Nixon and Crews, 2004). Differential effects on BrdU vs. Ki67 could also indicate cell cycle perturbation as we have observed during intoxication in adolescent male rats (Morris et al., 2010a; McClain et al., 2011). Next, in order to establish whether reactive neurogenesis occurs due to activation of NPCs in abstinence, we quantified the number of Ki67 and Sox2, an NPC marker, co-labeled cells within the SGZ of the dentate gyrus. Lastly, 14 days after the binge ethanol exposure, we investigated the expression of immature neurons as well as potentially aberrant, ectopic newborn neurons utilizing NeuroD1 IHC, a transcription factor that is transiently expressed in progenitors that have committed to a neuronal fate (Gao et al., 2009; McClain et al., 2014; Hayes et al., 2018). Finally, Prox1 expression, a post-mitotic marker for dentate gyrus granule cells (Iwano et al., 2012), was used to additionally identify potentially ectopic granule cells in the hilus and molecular layer of the dentate gyrus as we have observed previously in adolescent male rats (McClain et al., 2014).

#### 3,3′-Diaminobenzidine Tetrahydrochloride (DAB) IHC

All free-floating sections in a series were processed for IHC as previously described (McClain et al., 2011; Hayes et al., 2018). For Sox 2 and Ki67 T7 IHC, two control rats were dropped in both analyses due to problems with tissue integrity, with an additional two ethanol-treated rats removed from the Ki67 T7 analysis as a result of damage from slide breakage. For Sox2, Ki67, NeuroD1, and Prox1, every 12th section was washed in Tris-Buffered Saline (TBS) followed by incubation in 0.6% hydrogen peroxide for 30 min to quench endogenous peroxidases. Sections were then incubated in Antigen Retrieval Citra Solution (Biogenex, Freemont, CA, United States) at 65°C for 1 h. Next, a blocking buffer of 0.1% triton-X and 3% normal serum (as appropriate, Vector Laboratories, Burlingame, CA, United States) in TBS for 30 min to block non-specific binding. The sections were then incubated in blocking buffer with primary antibodies 24–48 h at 4°C (see Table 1 for antibody specifications). Following primary antibody incubation, tissue was washed in blocking buffer followed by incubation in species specific secondary antibodies (Table 1) and normal serum for 1 h at room temperature. Sections were then incubated in an avidin-biotin-complex (Vectastain Elite ABC kit, Vector Laboratories, Burlingame, CA, United States) for 1 h and developed with nickel-enhanced DAB (Vector Laboratories, Burlingame, CA, United States). Finally, sections were mounted on glass slides and coverslipped with Cytoseal^®^ (Thermo Fisher Scientific, Waltham, MA, United States).

**TABLE 1:** Antibody specifications.

Primary antibody	Company; Product no.	Conc.	Time in primary (h)	Secondary antibody (source)
**DAB**				
Goat anti-NeuroD1	R&D systems; AF2746	1:400	24	Biotinylated rabbit anti-goat (Vector)
Goat anti-Prox1	R&D systems; AF2727	1:200	24	Biotinylated rabbit anti-goat (Vector)
Mouse anti-BrdU	Millipore; MAB3424	1:4,000	24	Biotinylated horse anti-mouse (Vector)
Mouse anti-Ki67	Leica; NCL-L-Ki67-MM1	1:100	48	Biotinylated horse anti-mouse (Vector)
Rabbit anti-Sox2	Millipore; AB5603	1:1,000	24	Biotinylated goat anti-rabbit (Vector)
**Fluorescent**				
Mouse anti-Ki67	Leica; NCL-L-Ki67-MM1	1:100	48	AlexaFluor goat anti-mouse 555 (Invitrogen)
Rabbit anti-Sox2	Millipore; AB5603	1:1,000	48	AlexaFluor goat anti-rabbit 488 (Invitrogen)

#### Bromodeoxyuridine (BrdU) IHC

Immunohistochemistry procedures for BrdU detection were similar to those described in McClain et al. (2011), Hayes et al. (2018). For the T7 IHC, one control and two ethanol-treated rats were dropped from the T7 analysis. The control was for apparent BrdU injection failure, while the two ethanol-exposed rats were due to issues involving tissue integrity. Briefly, every 6th section in the series was used and incubated in 0.6% hydrogen peroxide for 30 min. DNA was then denatured using sodium citrate in 50% formamide at 60°C for 2 h. Next, sections were exposed to 2N HCl at 37°C for 1 h. Following neutralization of HCl with 0.1 M Boric Acid (pH 8.5), a blocking buffer consisting of 0.1% triton-X and 3% horse serum (Vector Laboratories, Burlingame, CA, United States) in TBS was used for 30 min to block non-specific binding. Sections were then washed in MgCl reaction buffer followed by incubation of DNase in reaction buffer for 1 h. Tissue was then incubated overnight in mouse anti-BrdU primary antibody in blocking buffer at 4°C. The following day, tissue was washed 3 times for 10 min each in blocking buffer, followed by incubation in a horse anti-mouse secondary antibody (Vector Laboratories, Billerica, MA, United States) for 1 h. Tissue was placed in an avidin-biotin-complex (ABC; Vector Laboratories, Burlingame, CA, United States) for an additional hour and stained with a nickel-enhanced DAB (Vector Laboratories, Burlingame, CA, United States). Finally, tissue was mounted to slides, allowed to dry, and sealed with Cytoseal^®^ (Thermo Fisher Scientific, Waltham, MA, United States) and a glass coverslip.

#### Dual Fluorescent IHC

Dual IHC labeling of Sox2 and Ki67 antibodies was used to examine the changes in NPC activation in the hippocampal SGZ during abstinence from alcohol. Four control rats were excluded from this analysis due to tissue integrity issues. Free-floating sections were processed as previously described (Hayes et al., 2018): every 12th section was washed in TBS, followed by 1 h of antigen retrieval in Citra Plus solution (Biogenex Laboratories, San Ramon, CA, United States) at 65°C. Sections were then placed in a blocking buffer of 10% normal goat serum, 0.1% triton-X, and TBS for 30 min and then incubated with anti-Sox2 and anti-Ki67 primary antibodies (see Table 1) in blocking buffer for 48 h at 4°C. Next, sections were rinsed in the blocking solution and incubated in species specific Alexa Fluor secondary antibodies (Table 1) with 1.5% normal goat serum for 1 h at room temperature. After final washes in TBS, sections were mounted onto glass slides, dried, and coverslipped with ProLong Gold Anti-fade medium (Invitrogen).

### Quantification

An unbiased profile counting methodology with 100% sampling fraction was utilized for each section in order to be identical to previous studies conducted in male rats (Crews et al., 2004; Noori and Fornal, 2011; Hayes et al., 2018). This approach was utilized as a result of the low cell number and/or heterogenous nature of the cell markers selected. Slides were coded so that experimenters were blinded to experimental conditions. The dorsal hippocampus of one hemisphere was quantified between Bregma −1.92 and −5.52 mm as determined by Paxinos and Watson, 2009 at either every 6th section (240 μm apart; BrdU) or every 12th section (480 μm apart; all other antibodies). This results in approximately 5–9 sections per subject (every 12th) or 10–16 sections per subject (every 6th). Immunoreactive cells in the SGZ (∼30 μm width or three granule cell bodies between the granule cell layer (GCL) and hilus) of the dorsal hippocampus were quantified identical to previous work (Hayes et al., 2018). For hilar and molecular layer ectopic cell counts, a region of 2–3 granule cell widths, respectively, (∼10 μm) outside of the GCL was analyzed (Liang et al., 2013; McClain et al., 2014). DAB-labeled counts were obtained using a 60× or 100× oil immersion objective and a 2× coupler on an Olympus BX43 or BX51 microscope (Olympus, Center Valley, PA, United States). For the quantification of anti-Sox2 and anti-Ki67 immunopositive cells and co-immunofluorescence, the Olympus BX51 microscope with a 40× lens at a 2× magnification was employed. Initially, Sox2+/Ki67+ cells were analyzed for Sox2+ expression and followed by assessment of Ki67+ expression. Secondarily, co-labeling was verified in several cells in representative tissue sections from each group on a Zeiss LSM 710 confocal microscope (Carl Zeiss Microscopy, LLC, White Plains, NY, United States; UT Microscopy and Flow Cytometry Core) with 0.5 μm Z-plane sections obtained with a 63× oil immersion lens and 3D rendered using Zen software (Carl Zeiss Microscopy, LLC, White Plains, NY, United States; UT Microscopy and Flow Cytometry Core).

### Statistical Analysis

All data were assembled in Excel (Microsoft Office v16) and analyzed and graphed using GraphPad Prism (version 9, GraphPad Software, La Jolla, CA, United States). All data are reported as mean ± SEM. Data were analyzed using Student’s *t*-tests (two-tailed) or Mann-Whitney *U* tests as appropriate for equal or unequal variance, respectively. BrdU and Ki67 utilized a two-way ANOVA with Bonferroni multiple comparisons. The relationship between the expression of all the neurogenesis markers used and BEC or withdrawal severity was analyzed using Pearson and Spearman correlations, respectively. Significance was accepted at *p* < 0.05.

## Results

### AUD Model Data

Adult, female rats underwent 4 days of binge alcohol exposure, parameters for which are detailed for each cohort in Table 2. Although the rat orders were identical (age 9–10 weeks), starting weights differed slightly between the T7 and T14 cohort (Supplementary Figure 1). Importantly, within each cohort there were no differences between the ethanol and control groups in initial weight prior to the start of the binge nor rate of weight loss across the binge. Overall, ethanol-treated rats averaged intoxication scores of 1.5 ± 0.1 (ataxia, with elevated abdomen), which produced a mean dose of 10.6 ± 0.3 g/kg/day and peak BECs of 344.2 ± 28.3 mg/dL as measured on the third day of the binge. Assessment of withdrawal behaviors began 10 h after their final dose of ethanol and lasted for 16 h. Ethanol-treated rats expressed average withdrawal scores of 0.7 ± 0.2 out of four (hyperactivity) with a peak score of 2.8 ± 0.3 (head tremors). While BEC and withdrawal scores appeared lower in the T14 cohort, only mean withdrawal was significantly different. All values in both cohorts were within the range of what has been reported for this model in male and female rats in our lab and others (Leasure and Nixon, 2010; Morris et al., 2010b; Barton et al., 2017; Maynard et al., 2017).

**TABLE 2:** Animal model data.

Time point	Subjects	Intox score	Dose (g/kg/day)	BEC (mg/dl)	Mean WD	Peak WD
T7	EtOH = 8 CON = 10	1.6 ± 0.1	10.2 ± 0.3	384.2 ± 20.8	1.0 ± 0.2	3.2 ± 0.2
T14	EtOH = 6 CON = 6	1.3 ± 0.2	11.2 ± 0.4	290.7 ± 55.4	0.3 ± 0.1*	2.2 ± 0.5

*p < 0.05 vs. T7.

### Ethanol Increases Cell Proliferation in the SGZ During Abstinence

**FIGURE 2:**
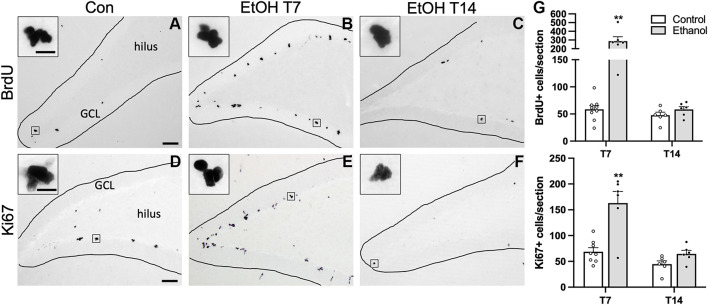
Cellular proliferation markers are increased following 7 or 14 days of abstinence in ethanol-treated rats. Representative images are shown for BrdU **(A–C)** and Ki67 **(D–F)** immunoreactivity in control [**(A)** BrdU: *n* = 9; **(D)** Ki67: *n* = 8], T7 ethanol-exposed [**(B)** BrdU: *n* = 6; **(E)** Ki67: *n* = 6), and T14 ethanol-exposed rats [**(C)** BrdU: *n* = 6; **(F)** Ki67: *n* = 6). **(G)** BrdU+ cells were counted throughout the SGZ and were increased significantly in the T7 ethanol-treated rats compared to controls and T14 ethanol-exposed rats. BrdU expression was confirmed with immunohistochemistry of the endogenous proliferation marker Ki67. As observed with the BrdU IHC, prior ethanol exposure increased KI67 expression in the SGZ following 7, but not 14 days of abstinence. Bars represent means ± SEM. Symbols represent individual data points. Scale bars = 100 μm; 10 μm inset. **p < 0.01.

In order to establish whether reactive neurogenesis occurs in females, we first examined two well-established proliferation markers in the SGZ, BrdU, a thymidine analog that was injected 2 h prior to perfusion, and Ki67, an endogenous marker of cell proliferation. In both controls and ethanol-exposed rats, BrdU+ cells were apparent throughout the SGZ in clusters, as expected (Figures 2A–C). We found significant main effects of group (*F*_1,23_ = 26.15, *p* < 0.0001) and time (*F*_1,23_ = 26.02, *p* < 0.0001), as well as a significant group by time interaction (*F*_1,23_ = 21.62, *p* = 0.0001). Multiple comparisons revealed significantly more BrdU+ cells in the T7 ethanol-exposed rats compared to both the T7 and T14 controls, as well as the T14 ethanol-treated rats (*p* < 0.0001 for all T7 comparisons). Next, we examined Ki67 immunoreactivity in an adjacent series of tissue as a means of confirming the exogenous cellular proliferation marker, BrdU, but also as a tool to screen for potential cell cycle effects revealed by differential effects on BrdU+ vs. Ki67+ cells in adolescent males (Morris et al., 2010a; McClain et al., 2011). Qualitatively, Ki67 immunoreactivity shows a similar pattern of clustered cells along the SGZ, as observed with BrdU (Figures 2D–F). Overall immunoreactive profiles for Ki67 appear lower than what was observed with a prior manufacturer. However, similar to BrdU immunoreactivity, we observed significant main effects of group (*F*_1,22_ = 25.06, *p* < 0.0001) and time (*F*_1,22_ = 21.78, *p* = 0.0001), as well as a significant group by time interaction (*F*_1,22_ = 9.22, *p* = 0.0061). Multiple comparisons revealed significantly more Ki67+ cell in the T7 ethanol-treated group compared to all other groups (*p* < 0.0001 for all T7 comparisons). Furthermore, correlational analysis revealed no significant relationship between either BrdU or Ki67 expression and BEC as well as observed withdrawal behaviors (Table 3).

**TABLE 3:** Correlations with BEC and withdrawal scores.

Measurement			BEC (mg/dl)		Mean WD		Peak WD	
			
		*n*	Pearson r	*P*-value	Spearman r	*P*-value	Spearman r	*P*-value
SGZ	T7 BrdU	6	0.33	0.52	0.14	0.80	−0.12	0.81
	T14 BrdU	6	−0.12	0.82	0.26	0.59	0.43	0.42
	T7 Ki67	6	0.36	0.48	0.32	0.56	0.32	0.51
	T14 Ki67	6	−0.55	0.23	−0.15	0.77	−0.20	0.71
	T7 Sox2	8	−0.24	0.57	−0.57	0.14	−0.69	0.07
	T7 Sox2/Ki67	8	0.22	0.61	−0.20	0.63	−0.35	0.39
GCL	T14 NeuroD1: Inner 1/3	6	0.06	0.91	0.44	0.38	−0.23	0.66
	T14 NeuroD1: Outer 2/3	6	−0.18	0.73	−0.07	0.90	−0.77	0.07
ML	T14 NeuroD1	6	0.48	0.34	0.42	0.41	−0.29	0.58
Hilus	T14 NeuroD1	6	0.51	0.31	0.70	0.12	−0.03	0.95
ML	T14 Prox1	6	0.34	0.51	−0.09	0.85	−0.26	0.66
Hilus	T14 Prox1	6	0.43	0.40	0.03	0.99	−0.37	0.50

### Ethanol Exposure Increases the Number of NPC Into Active Cycling

**FIGURE 3:**
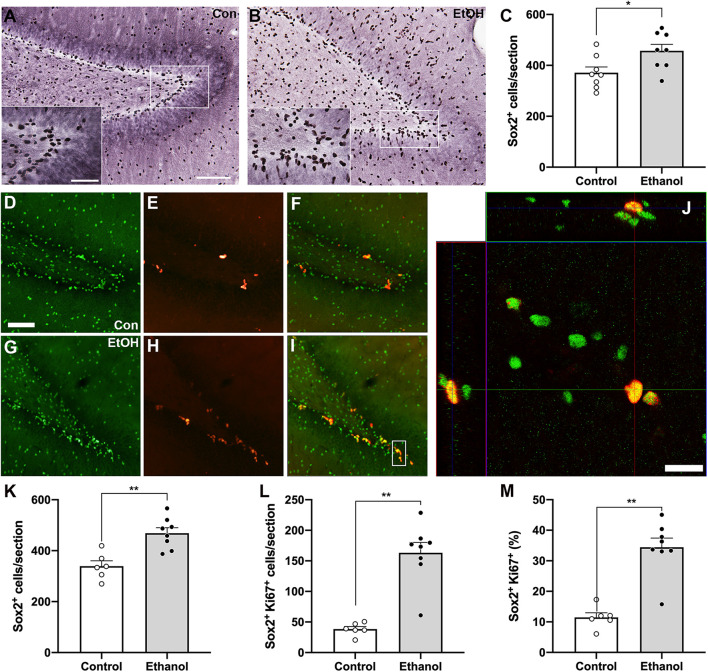
Alcohol-induced reactive neurogenesis is a result of an increase in active division of NPC. Representative images of Sox2+ cells in the hippocampus of a control [**(A)** *n* = 8] and ethanol-treated [**(B)** *n* = 8] rat at T7 as quantified in panel **(C)**. Representative images of Sox2+ **(D,G)**, Ki67+ **(E,H)**, and dual-labeled cells **(F,I)** in a control **(D–F)**, *n* = 6) and an ethanol-treated [**(G–I)** *n* = 8] rat following 7 days of abstinence. **(J)** 3-D rendered z-stack images “orthogonal view” to show Sox2+/Ki67+ co-labeling in the same cell. **(K)** The increase in Sox2 expression was replicated with immunofluorescence. Additionally, all Sox2+ cells were assessed for the presence of Ki67 expression. As previously observed in males, there was a significant increase in the amount **(L)** and proportion **(M)** of actively dividing NPC in ethanol-treated rats compared to controls at T7. **(M)** Data are mean ± SEM. Symbols represent individual data points. Scale bars = 100 μm in A main, D; 50 μm A inset; 20 μm in panel **(J)**. *p < 0.05; **p < 0.01.

Next, we examined NPC activation by quantifying the number of Ki67 and Sox2 co-labeled cells, as we observed an increase in activated NPCs in the SGZ of males following alcohol dependence (Hayes et al., 2018). First, we quantified Sox2+ cells, a NPC marker, with DAB IHC within the SGZ 7 days post 4-day binge exposure (Figures 3A,B), which revealed a significant 23% increase in Sox2+ cells of ethanol-treated animals compared to controls [Figure 3C; *t*(14) = 2.5, *p* = 0.025]. Next, in order to examine whether alcohol altered the number of actively cycling Sox2+ progenitors in the SGZ, the number of Sox2+ cells that also expressed Ki67 was quantified. We found a significant increase in the number of Sox2+ cells that co-expressed Ki67 in the ethanol-treated females compared to controls (Figure 3L; *U* = 0, *p* = 0.0007). However, this increase in the amount of colabeling may be the result of a greater amount of Sox2+ and Ki67+ cells in the DG of our ethanol-treated females. Therefore, to see if the increase in the amount of double labeled cells reflect changes in active cycling, we examined alterations in the proportion of Sox2+ cells that also express Ki67. In ethanol-treated females, 34% of all Sox2+ cells in the SGZ were also Ki-67+, whereas only 11.5% were in controls (Figure 3M; *p* < 0.05). These increases in NPC expression and activation were not correlated to withdrawal scores or BEC (Table 3).

### Prior Ethanol Exposure Increases the Number of Newly Differentiated Neurons in the SGZ

**FIGURE 4:**
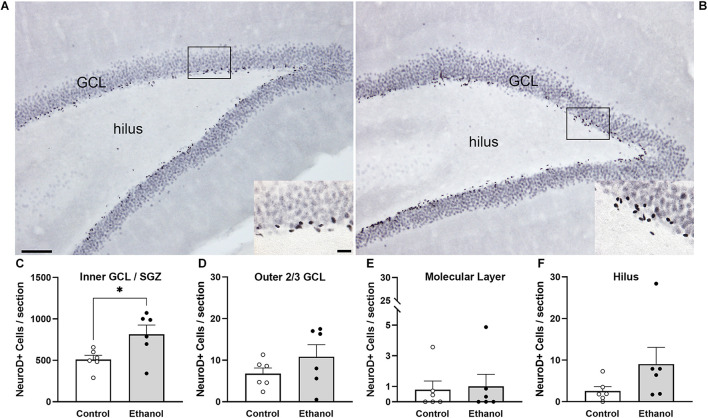
NeuroD1 expression is increased in neurogenic regions in ethanol-treated rats following 14 days of abstinence. Representative images of NeuroD1 immunoreactivity in a control [**(A)** *n* = 6] and an ethanol-exposed [**(B)** *n* = 6] rat at T14. Expression of NeuroD1 was increased in the SGZ and inner third of the GCL **(C)**, but not the outer 2/3 **(D)**, molecular layer **(E)**, or hilus **(F)**. Bars represent means ± SEM. Symbols represent individual data points. Scale bars = 100 μm; 20 μm inset. *p < 0.05.

NeuroD1 is normally seen in the SGZ and inner 1/3 of the GCL as immature neurons migrate following cell division, as is visible in both groups (Figure 4). We observed a significant increase in NeuroD1+ cells in the inner 1/3 of the GCL/SGZ in ethanol-treated female rats at T14 compared to controls [Figure 4C; *t*(10) = 2.5, *p* = 0.03]. In order to probe for a relationship between reactive neurogenesis and alcohol dependence as has been observed in males (Nixon and Crews, 2004), correlations were conducted between NeuroD1 profile counts and measures of withdrawal severity (peak/mean withdrawal scores) or BEC (Table 3). No significant relationship between NeuroD1+ cell counts and these aspects of alcohol dependence were observed.

### Ethanol Exposure Does Not Produce Ectopic Granule Cells in the Dentate Gyrus

**FIGURE 5:**
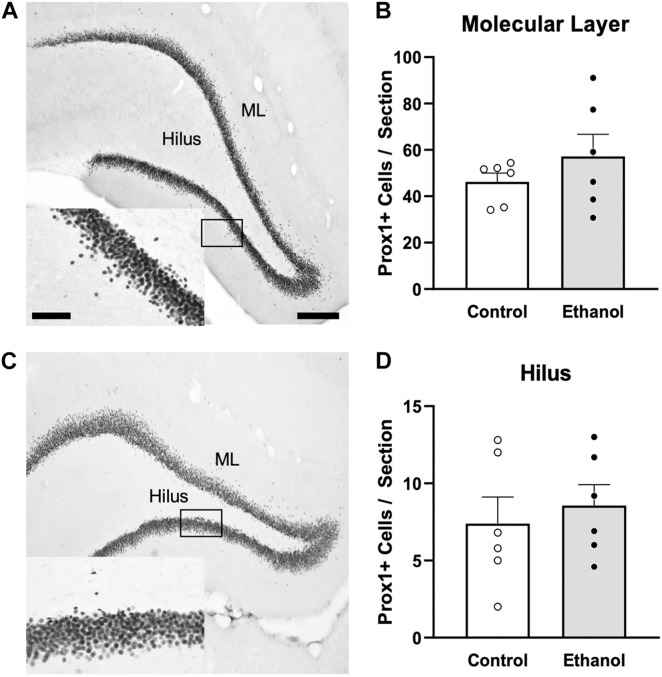
Ectopic granule cells were not observed in the DG of female rats 14 days following ethanol exposure. Representative images show Prox1 immunoreactivity in a control [**(A)** *n* = 6] and ethanol-treated [**(C)** *n* = 6] rat. Prior alcohol exposure had no impact on Prox1+ ectopic cells in the molecular layer **(B)** or hilus **(D)** of adult female rats. Data are mean ± SEM. Symbols represent individual data points. Scale bars = 200 μm; 60 μm inset.

In order to investigate whether ectopic neurogenesis is occurring in adult female rats after alcohol dependence, NeuroD1+ cells were also quantified in the outer 2/3 of the GCL, the hilus, and the molecular layer of the dentate gyrus at T14. Analysis of the outer 2/3 of the GCL [Figure 4D; *t*(10) = 1.3, *p* = 0.24], molecular layer [Figure 4E; *t*(10) = 0.03, *p* = 0.98], and hilus [Figure 4F; *U* = 7.5, *p* = 0.1] did not reveal significant differences in the number of NeuroD1+ cells in any of those regions between groups. Prox1 expression was also examined for evidence of ectopic cells. As with NeuroD1+ analysis, we observed no significant differences in the number of Prox1+ cells in ethanol-treated females compared to controls in both the molecular layer and hilus (Figure 5, molecular layer: *t*(10) = 1.069, *p* = 0.31; hilus: *t*(10) = 0.5341, *p* = 0.61). As ectopic neurogenesis occurs with seizures and the number of ectopic Prox1+ cells in the molecular layer appeared more variable in the ethanol-treated rats, we further probed the data for a potential relationship to withdrawal severity as well as other aspects of the model. For both the hilus and molecular layer, ectopic Prox1+ cell number did not correlate with either measure of withdrawal severity (peak/mean withdrawal scores) or BEC (Table 3).

## Discussion

The impact of various types of alcohol exposures on adult neurogenesis has been well-studied in male rodents, however, there has been a considerable lack of research on the effect in females alone. Therefore, in this study, we sought to investigate the effects of binge ethanol exposure on the phenomena of reactive neurogenesis in the hippocampus of female rats. Following 7 days of abstinence from alcohol dependence, we observed a striking fourfold increase in NPC proliferation (BrdU+ and Ki67+), which is likely due to an increase in active cycling of NPCs (Sox2+ /Ki67+). Seven days later at T14, newborn neurons indicated by NeuroD1+ immunoreactivity were similarly increased. Furthermore, this rebound effect did not appear to produce an increased number of ectopic neurons (ectopic Prox1+ or NeuroD1+ cells) as occurs in seizure models or in adolescent male rats exposed to alcohol (McClain et al., 2014). Taken together, these results suggest that alcohol dependence results in reactive, adult neurogenesis in females. Although qualitative and not a direct comparison, reactive neurogenesis appears similar between adult males and females after the 4-day binge (Nixon and Crews, 2004; Hayes et al., 2018), however, some subtle differences were observed in this study compared to our past work and may warrant future direct comparisons between the sexes.

The neuroplasticity or damage that occurs with alcohol dependence results in a transient increase in the generation of new neurons, a phenomenon that has been termed reactive neurogenesis when it occurs after an insult (Molowny et al., 1995; Nixon and Crews, 2004). Binge ethanol exposure in adult females resulted in increases in NPC proliferation according to BrdU and Ki67 IHC (Figure 2). While the mechanism for this reactive neurogenesis in females is under investigation, our report in males showed that these changes in proliferation were not the result of alterations in the cell cycle but increased NPC activation (Hayes et al., 2018). Although changes in cell cycle dynamics were not examined in the current study, the similar increases in Ki67+ and BrdU+ proliferating cells suggest that the cell cycle is not likely perturbed by alcohol exposure at this time point (McClain et al., 2011). Furthermore, the increased recruitment of NPCs into active cycling in females (Ki67+/Sox2+ cells) may suggest a similar mechanism underlying alcohol-induced reactive neurogenesis as found in males (Hayes et al., 2018). Yet further analysis is still required as a limitation of this work is that Sox2 is not specific to progenitors and can also be expressed in astrocytes (Komitova and Eriksson, 2004). However, in the current study, Sox2-Ki67 co-labeling was only examined in the SGZ where progenitors reside and astrocyte proliferation has not been observed in models of AUD, thus supporting the interpretation that proliferating Sox2+ cells are most likely NPCs. Though only qualitative, in the current study, we observed an increase in NPC activation in females (Figure 3). Females exhibited twice the amount (∼30%) of activated progenitors at this time point than that previously seen in males (∼15%; Hayes et al., 2018). While not a direct comparison between the sexes, this observation is surprising given that most studies see enhanced survival of newborn neurons in females and not differences in proliferation (see Mahmoud et al., 2016 for review). This differential increase in activated progenitors is intriguing considering the higher baseline levels of cellular proliferation in the dentate gyrus of males compared to females under normal conditions (Yagi et al., 2020). Circulating gonadal hormones have been implicated in changes in cellular proliferation in the hippocampus. Higher levels of proliferating cells are found in females in the proestrus stage, or when concentrations of estradiol are highest, compared to males and females in other estrous cycle stages (Tanapat et al., 1999). Furthermore, repeated estradiol administration in gonadectomized rats resulted in increased cell proliferation in the SGZ of females but not males (Barker and Galea, 2008). As previously stated, we used intact female rats, but did not monitor estrous stage throughout the experiment. Although we cannot rule out the possibility of an interaction between hormone levels and alcohol exposure on components of adult neurogenesis, prior preclinical studies found that estrous stage had no impact on binge alcohol-induced hippocampal damage or recovery (Maynard and Leasure, 2013; Maynard et al., 2017). While changes in circulating hormones associated with estrous cycle garner much of the focus, androgens have also been found to increase adult neurogenesis *via* the androgen receptor pathway in young adult males but not females (Duarte-Guterman et al., 2019). Thus, despite both sexes exhibiting reactive, adult neurogenesis, it remains possible that the mechanism underlying this process differs between the sexes and warrants future investigation.

In order to identify and quantify the relative change in the number of immature neurons in females as a result of alcohol-induced reactive neurogenesis, we used an immature neuronal marker, the transcription factor NeuroD1 (Gao et al., 2009). Consistent with our previous studies in males, we found significantly more cells committed to the neuronal fate born from alcohol-induced reactive neurogenesis in the ethanol-treated females compared to controls at day 14 of abstinence (Nixon and Crews, 2004; Hayes et al., 2018). While there was still a significant increase in NeuroD1+ cells in the ethanol-treated females at day 14 in the current study, when compared to our previous studies in males, we observed fewer NeuroD1+ cells in the dentate gyrus despite more progenitors activated into proliferation. While these differences in immature neurons could be attributed to differences in the source of antibodies used, it is possible that circulating hormones may influence alcohol-induced proliferating cell survival.

Teasing apart the precise mechanisms of reactive neurogenesis after alcohol dependence remains a key area of inquiry. During the development of dependence, multiple events occur as part of alcohol’s neuroplastic effects on the brain that overlap with mechanisms known to cause reactive neurogenesis (reviewed in Nixon, 2006; Geil et al., 2014). Specifically, cell death and degeneration, a hyperglutamatergic state, and alcohol withdrawal seizures are mechanistically intertwined and also the major causes implicated in reactive neurogenesis (Nixon and Crews, 2004; Crews and Nixon, 2009; Mandyam, 2013; Geil et al., 2014). Though the role of seizures in reactive NPC proliferation had been ruled out at least in males (e.g., Nixon and Crews, 2004), recent studies bring to light that newborn neurons may be driving the development of alcohol withdrawal seizures (Lee et al., 2019). This latter point strongly suggests that it is the maladaptive plastic events such as deranged glutamatergic signaling or neuroimmune activation that occur during the development of dependence that underlies these intertwined phenomena (Crews and Nixon, 2009; Avchalumov et al., 2021). This remains speculative as what little is known about cellular mechanisms underlying alcohol-induced reactive neurogenesis jumps forward to implicate brain-derived neurotrophic factor (BDNF) in the hippocampus (McClain et al., 2014; Somkuwar et al., 2016; Hayes et al., 2018). Thus, while a hyperglutamatergic state, seizures, and neuroimmune activation can be linked through BDNF, much remains to be discerned regarding the involvement of this cascade in mechanisms of reactive, adult neurogenesis whether in males or females.

Adult neurogenesis is essential for hippocampal structure and function, with increases associated with improved cognitive performance (Sahay et al., 2011). As hippocampal neurodegeneration is a consequence of alcohol dependence, reactive neurogenesis has been implicated in repopulating the hippocampal dentate gyrus in males (Mandyam and Koob, 2012; Gage and Temple, 2013). However, the functional importance of this reactive increase remains up for debate. The increased production of newborn neurons coincides with behavioral recovery on performance of the Morris Water Maze task, a hippocampal-dependent, learning and memory task (Nickell et al., 2020; Nawarawong et al., 2021). In addition, cells born during reactive neurogenesis appear to be incorporated into hippocampal circuitry. Recent studies in male rats have observed similar expression levels of the immediate early gene c-Fos in newborn neurons in both ethanol-treated rats and controls (Nawarawong et al., 2021). However, a recent human study found that females demonstrated prolonged cognitive impairment due to a slower recovery from alcohol compared to males (Luquiens et al., 2019). Furthermore, a chronic model of alcohol exposure in female mice found that the alcohol-induced reactive increase in neurogenesis resulted in aberrant hippocampal neuronal integration and cognitive dysfunction (Golub et al., 2015). While we did not study cognitive impairment in the current study, we did not observe any differences in ectopic neurogenesis (Figures 4, 5). While there are a number of experimental differences between our rat study and Suh and colleague’s mouse studies (Golub et al., 2015; Lee et al., 2019), much remains to be learned about how cells born during reactive neurogenesis function, such as whether they are incorporated into neuronal ensembles and their contribution to the overall integrity of the hippocampus. Future studies understanding what underlies these changes in females as well as those directly comparing both sexes are necessary to fully understand the role and functional implications of reactive neurogenesis in the recovery of hippocampal structure and function in models of AUD.

A limitation of the current study is the lack of a full time course assessing the various components of adult neurogenesis, as has previously been performed in males (Nixon and Crews, 2002, 2004; Hayes et al., 2018). Under normal conditions, hippocampal neuronal maturation is slower in females compared to males (Mahmoud et al., 2016; Yagi et al., 2020). The time points used in the current study are based on studies performed in males, therefore it is possible that the time course of proliferation effects may differ in females from what has previously been reported in males (Nixon and Crews, 2004; Hayes et al., 2018). Furthermore, the current study only examined an immature neuronal marker (NeuroD1). While cells that survive to 14 days are thought to persist long-term and are incorporated into hippocampal circuity (Kempermann et al., 2003; Snyder and Cameron, 2012), sex differences in the number of immature neurons in the dentate gyrus have been observed, with increased immature neuron numbers in females under normal conditions (Siddiqui and Romeo, 2019). In depth assessments of adult neurogenesis have not been examined extensively in females alone and may differ or interact with the sex differences in reaction to alcohol dependence (Siddiqui and Romeo, 2019; Yagi and Galea, 2019). Furthermore, any interpretations must take into consideration sex differences in hippocampal volume and makeup. Previous studies have observed larger GCL volumes and a greater density of NPCs in the dorsal hippocampus of males compared to females (Duarte-Guterman et al., 2019; Yagi et al., 2020). As an available pool of NPCs is a requirement of reactive neurogenesis (Yu et al., 2008; Blaiss et al., 2011; Sun et al., 2013), basal differences in populations of progenitors between the sexes may explain the greater impact of the neurotoxic effects of alcohol on females. Taken together, this suggests that alcohol’s damaging effects may be where the sexual dimorphism lies (Wilhelm et al., 2016; Cortez et al., 2020) rather than in reactive proliferation. Perhaps this is why there is not a relationship between BrdU+ cell number and withdrawal severity (Table 3) as there is in males (Nixon and Crews, 2004). Indeed, there are known sex differences in several events surrounding alcohol-induced damage, such as neuroimmune activation (Wilhelm et al., 2016; Barton et al., 2017; Rath et al., 2020), effects of which notably also interact with the control of adult neurogenesis (Alvarez Cooper et al., 2020; Diaz-Aparicio et al., 2020).

An important consideration when investigating the effects of alcohol in females is potential differences in alcohol pharmacokinetics. Compared to males, females have a faster rate of absorption, distribution to the brain, and elimination of alcohol (Robinson et al., 2002; Cederbaum, 2012). However, in preclinical models, these differences are not thought to be influenced by the fluctuating hormone levels associated with the different estrous stages (Robinson et al., 2002; Maynard et al., 2017). The binge model of exposure was chosen specifically to circumvent potential alcohol pharmacokinetic differences as dosing is based on intoxication behavior (Majchrowicz, 1975). This binge model has been useful in comparing across ages where slight pharmacokinetic differences have occurred (e.g., Varlinskaya and Spear, 2004; Morris et al., 2010b) or within drug discovery studies (Liput et al., 2013). Furthermore, similar BECs have been observed for both males and females with this model (Maynard et al., 2017; West et al., 2018). Therefore, with the consistency in intoxication levels between the sexes in the binge model of alcohol exposure, any differences that emerge in females would be the result of alternative mechanisms and not a direct result of pharmacokinetics differences.

While the binge model of alcohol exposure in preclinical studies reduces potential sex differences in pharmacokinetics, potential differences in withdrawal behaviors, and severity are unavoidable. In clinical alcohol dependent populations, men report more withdrawal symptoms and more impaired general functioning compared to females (Deshmukh et al., 2003). This is mirrored in preclinical studies that have observed quicker recovery from alcohol dependence in females (Devaud and Chadda, 2001; Alele and Devaud, 2007). In the current study, average withdrawal behaviors were similar to slightly less severe than those previously reported in males (Hayes et al., 2018). While a direct comparison is necessary to flesh out quantitative changes, alterations in GABAergic inhibitory tone have been implicated in the sex differences in withdrawal severity (for review see Sharrett-Field et al., 2013). Devaud and Alele (2004) reported greater levels of alpha subunits of the GABAa receptor in males, while they observed increased beta subunit expression in females. These alterations in GABA receptor subunits result in decreased inhibitory tone in males and may underlie the more prolonged and severe observed withdrawal behaviors. A common withdrawal behavior is seizures, or spontaneous convulsions. In seizure models, seizure behavior is correlated with increased ectopic cellular expression. In adolescent males, we observed an increase in ectopic cells that was positively correlated with withdrawal severity. However, in this current study utilizing a binge model of alcohol dependence in females, seizure expression did not alter aberrant cellular division. Furthermore, the lack of changes we observed in ectopic Prox1 expression suggests an underlying neuroprotective mechanism that may play a role in differences in withdrawal behavior expression. Thus future work is required to understand the influence of sex on alcohol withdrawal and recovery.

## Conclusion

In conclusion, these data add to a nascent but growing body of work on the effects of alcohol exposure in females within the context of an AUD, as recent reviews have detailed (Cortez et al., 2020; Fama et al., 2020; White, 2020). Despite a greater vulnerability for alcohol-induced damage in females, the current study agrees with prior preclinical reports suggesting that females exhibit pronounced hippocampal structural recovery (Jacobson, 1986; Deshmukh et al., 2003; Leasure and Nixon, 2010; Maynard and Leasure, 2013; West et al., 2019). Following binge ethanol exposure, we found increased levels of cellular proliferation, activated NPCs, and immature neurons in the DG of females, phenomena consistent with increased adult neurogenesis potentially contributing to recovery. Whether such recovery mechanisms are sufficient to overcome prior damage (e.g., West et al., 2019) and the signaling mechanism behind these effects are the next questions. While these alterations in cellular proliferation and survival appear to be important to the structural and functional recovery of the hippocampus in males (Nickell et al., 2020; Nawarawong et al., 2021), little is known about the functional implications of these newborn cells in females. Further understanding of the role reactive neurogenesis plays in hippocampal recovery will allow for the identification of novel pharmacological targets for the therapeutic treatment of AUDs.

## Data Availability Statement

The raw data supporting the conclusions of this article will be made available by the authors, without undue reservation.

## Ethics Statement

The animal study was reviewed and approved by the University of Kentucky IACUC and The University of Texas at Austin IACUC.

## Author Contributions

HP and KN designed the experiments. NN, KT, SG, KN, HP, and CA performed the experiments. NN and KT performed the statistical analyses. NN and KN wrote the original draft of the manuscript and interpreted the results. NN, KT, CA, and KN prepared the figures. All authors have read, commented, edited, and agreed to the manuscript.

## Conflict of Interest

The authors declare that the research was conducted in the absence of any commercial or financial relationships that could be construed as a potential conflict of interest.

## Publisher’s Note

All claims expressed in this article are solely those of the authors and do not necessarily represent those of their affiliated organizations, or those of the publisher, the editors and the reviewers. Any product that may be evaluated in this article, or claim that may be made by its manufacturer, is not guaranteed or endorsed by the publisher.
