# Supplementary material for: Reactive, Adult Neurogenesis From Increased Neural Progenitor Cell Proliferation Following Alcohol Dependence in Female Rats
Source: Front Neurosci. 2021 Sep 14;15:689601. doi: 10.3389/fnins.2021.689601 (PMC8477003; doi:10.3389/fnins.2021.689601)
Supplement: Supplementary file 1 [file Image_1.pdf]

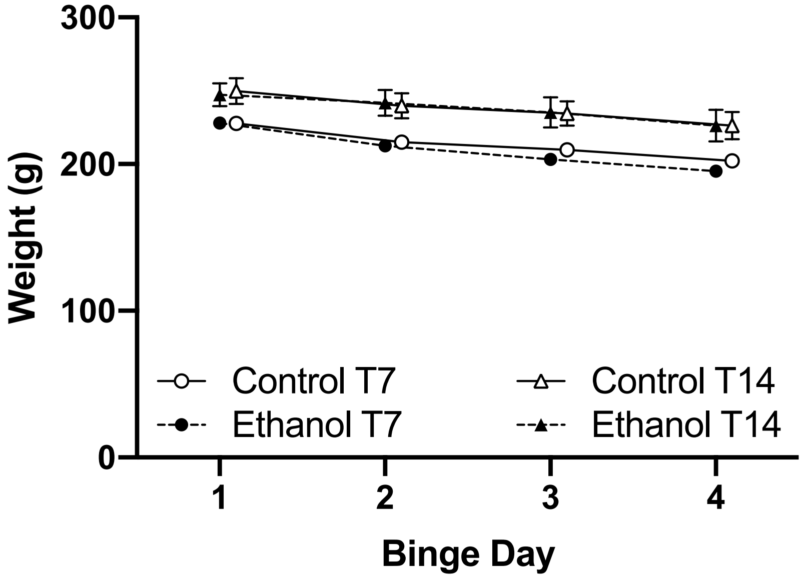

Supplemental Figure 1: Regardless of ethanol exposure, binge procedures result in weight loss for all groups. A three-way repeated measures ANOVA found significant main effects of binge day ($p<0.0001$) and abstinence time point ($p<0.0001$), but not ethanol exposure. As there were differences in starting weights between the T7 and T14 abstinence time points, but a similar weight loss trajectory for all groups, we observed a binge day by abstinence time point interaction ($p=0.0001$). Furthermore, there was a binge day by abstinence time point by ethanol exposure interaction ($p=0.03$). Bonferroni corrections revealed no significant differences in weight between the control and ethanol groups within abstinence time points across the binge days.
